# Baseline gut microbiota diversity and composition and albendazole efficacy in hookworm-infected individuals

**DOI:** 10.1186/s13071-024-06469-1

**Published:** 2024-09-12

**Authors:** Javier Gandasegui, Pedro E. Fleitas, Paula Petrone, Berta Grau-Pujol, Valdemiro Novela, Elisa Rubio, Osvaldo Muchisse, Anélsio Cossa, José Carlos Jamine, Charfudin Sacoor, Eric A. T. Brienen, Lisette van Lieshout, José Muñoz, Climent Casals-Pascual

**Affiliations:** 1 Barcelona Institute for Global Health, Hospital Clínic-Universitat de Barcelona, Barcelona, Spain; 2 Manhiça Health Research Centre (CISM), Manhiça City, Mozambique; 3 Mundo Sano Foundation, Buenos Aires, Argentina; 4 Department of Clinical Microbiology (CDB), Hospital Clínic-University of Barcelona, Barcelona, Spain; 5 Leiden University Center for Infectious Diseases, Parasitology Research Group, Leiden University Medical Center (LUMC), Leiden, The Netherlands

**Keywords:** Microbiota, Hookworm, Albendazole, Treatment response

## Abstract

Soil-transmitted helminth (STH) infections account for a significant global health burden, necessitating mass drug administration with benzimidazole-class anthelmintics, such as albendazole (ALB), for morbidity control. However, ALB efficacy shows substantial variability, presenting challenges for achieving consistent treatment outcomes. We have explored the potential impact of the baseline gut microbiota on ALB efficacy in hookworm-infected individuals through microbiota profiling and machine learning (ML) techniques. Our investigation included 89 stool samples collected from hookworm-infected individuals that were analyzed by microscopy and quantitative PCR (qPCR). Of these, 44 were negative by microscopy for STH infection using the Kato-Katz method and qPCR 21 days after treatment, which entails a cure rate of 49.4%. Microbiota characterization was based on amplicon sequencing of the V3–V4 16S ribosomal RNA gene region. Alpha and beta diversity analyses revealed no significant differences between participants who were cured and those who were not cured, suggesting that baseline microbiota diversity does not influence ALB treatment outcomes. Furthermore, differential abundance analysis at the phylum, family and genus levels yielded no statistically significant associations between bacterial communities and ALB efficacy. Utilizing supervised ML models failed to predict treatment response accurately. Our investigation did not provide conclusive insights into the relationship between gut microbiota and ALB efficacy. However, the results highlight the need for future research to incorporate longitudinal studies that monitor changes in the gut microbiota related to the infection and the cure with ALB, as well as functional metagenomics to better understand the interaction of the microbiome with the drug, and its role, if there is any, in modulating anthelmintic treatment outcomes in STH infections. Interdisciplinary approaches integrating microbiology, pharmacology, genetics and data science will be pivotal in advancing our understanding of STH infections and optimizing treatment strategies globally.

**Graphical Abstract:**

**Figure:**
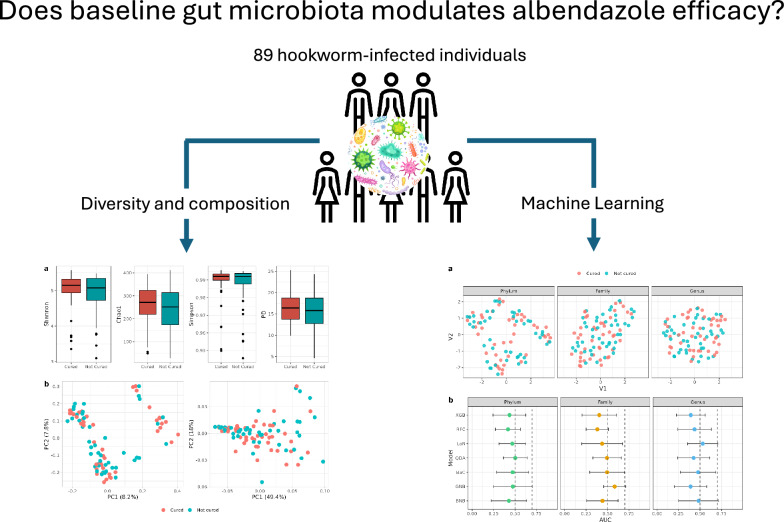

**Supplementary Information:**

The online version contains supplementary material available at 10.1186/s13071-024-06469-1.

## Background

Soil-transmitted helminths (STH) are a group of parasitic worms that cause a major cumulative burden of disease worldwide. The WHO estimates that around 1.5 billion people are infected with at least one of four STH species, including *Ascaris lumbricoides*, *Trichuris trichiura* and the two hookworms *Necator americanus* and *Ancylostoma duodenale* [1]. WHO guidelines for STH infections call for controlling morbidity through mass drug administration (MDA) of the benzimidazole-class anthelmintics albendazole (ALB) or mebendazole—mainly to preschool-aged children, school-aged children and women of reproductive age [2]. However, benzimidazole-based MDA is not equally effective against all STH species, with lower efficacy against *T. trichiura*, medium efficacy against hookworms and higher efficacy against *A. lumbricoides* [3].

In addition to the variability in benzimidazole efficacy among STH species, there is a high variability in benzimidazole response between individuals infected with *T. trichiura* or hookworms. A recent clinical trial showed cure rates (CR) ranging from 56% to 96% between countries using ALB monotherapy for the treatment of hookworm infections [4]. Currently, there is no single comprehensive explanation for the observed variability in ALB treatment outcomes among individuals within the same geographical area, nor for the differing CR reported across study sites. One of the leading hypotheses is that the pharmacokinetics of ALB varies significantly between individuals, potentially influencing the clearance of parasites post-treatment [5, 6]. Another theory postulates that genetic factors within the parasite population may confer resistance to the drug, possibly exacerbated by prolonged MDA with benzimidazoles [7]. However, to date, none of these hypotheses have been conclusively substantiated in the context of STH infections.

In recent years, various studies have shown the effect of the gut microbiome on drug safety and efficacy and, consequently, the gut microbiome has been pinpointed as a factor that can also influence the efficacy of anthelmintics. The microbial community in the gut can modify the pharmacodynamics or activity of a medication either by directly transforming the drug or by altering the host’s metabolism or immune system [8–10]. The term pharmacomicrobiomics has been proposed to describe the influence of compositional and functional variations of the microbiome on drug efficacy and toxicity, thus contributing to an individual’s response to a specific drug [11]. To date, two studies have specifically focused on examining the possible influence of the baseline gut microbiome on the efficacy of anthelmintics in STH infections [12, 13], with each study reporting different findings. One of these studies found an association between the gut microbiota and the efficacy of combination therapy with ALB + ivermectin, whereas treatment outcome with ALB monotherapy did not correlate with specific microbial communities [12]. However, the analysis conducted in that study was based on enterotypes, limiting the broad extrapolation of its findings to different contexts. The second study did find some microbial communities within the human gut to be a potential indicator of ALB treatment failure in hookworm infection [13].

The study reported here builds upon two previous studies conducted by our team in which we assessed the prevalence of STH infections at the community level across Manhiça district, Mozambique [14], and then evaluated the treatment response with ALB in participants with STH infections using microscopy and quantitative PCR (qPCR) [15]. We also assessed the presence and frequency of putative benzimidazole resistance single nucleotide polymorphisms (SNPs) in the beta tubulin gene of *T. trichiura* and hookworms, but found no association with ALB efficacy, suggesting that other mechanisms are likely driving the variability in response to anthelmintics in the study area. We also explored whether the diversity and the composition of the baseline gut microbiota at the time of drug intake is associated and/or can predict the treatment response to ALB in hookworm-infected individuals. Using a robust diagnostic framework, we have combined conventional statistics with predictive analysis based on machine learning (ML) approaches to assess the relationship between the microbiota and ALB efficacy. We excluded participants infected only with *T. trichiura* and *A. lumbricoides* from this study due to the extremely low and extremely high CR for these species, respectively, observed in our previous study [15], as this would limit the comparison between patients who were cured and those who were not cured.

## Main findings

A total of 101 stool samples were collected from hookworm-infected individuals shortly before the initiation of ALB treatment. Complete baseline microbiota data were obtained from 89 individuals, of whom 51 (57.3%) and 38 (42.7%) were female and male, respectively, with a mean age of 30 (range 5–88) years. Hookworm presence in the stool had been previously determined by the Kato-Katz thick smear method and qPCR prior to the initiation of ALB treatment and 21 days after treatment initiation. A complete description of the study design and sample collection and processing has been described in our earlier studies [14, 15]. All included participants were diagnosed as being infected with hookworms by the Kato-Katz method and/or qPCR before treatment, whereas the outcome was defined as ‘cured’ based on the absence of hookworms after treatment, which means a negative result by the Kato-Katz method and qPCR 21 days after providing ALB. Of the 89 participants at baseline, 71 were STH positive by the Kato-Katz method, with a mean eggs per gram of stool (EPG) of 142.3 (range 12–1518) EPG, whereas all 89 participants were positive for STH by qPCR. After treatment, 45 participants were positive for STH by microscopy or qPCR, which indicates a CR of 49.4%; of these 45 participants, 13 were positive by the Kato-Katz method (mean and range EPG: 111.7 and 6–474, respectively) and 43 were positive by qPCR, with positivity detected in 11 samples by both methods. *Necator americanus* was the only hookworm species identified by qPCR. As expected, the inclusion of a molecular test complementing the microscopy-based diagnostic improved the identification of hookworm-infected individuals, particularly after treatment when the infection intensity was lower. We also detected 32 additional treatment failures by qPCR, thereby contributing to a more accurate assessment of ALB efficacy, which was not accomplished in previous studies [12, 13].

For microbiota characterization, we extracted DNA from stool samples using the PureLink™ Microbiome DNA Purification kit (Invitrogen™, Thermo Fisher Scientific, Waltham, MA, USA) according to the manufacturer’s instructions. DNA concentration and quality were measured with a Qubit 3.0 fluorometer (Life Technologies Europe, Ledeberg, Belgium). We then selected the V3–V4 region from the 16S ribosomal RNA (rRNA) gene (expected amplicon size: approximately 460 bp), and we used the primer pair described in the MiSeq™ rRNA Amplicon Sequencing protocol (Illumina, Inc., San Diego, CA, USA) as previously reported [16]. Sequencing was performed on an Illumina MiSeq platform according to manufacturer's specifications to generate paired-end reads of 300 base-length (Illumina, Inc.). Sequencing data were then processed using QIIME2 [17]. Amplicon sequencing data were obtained as a single file per run and per participant and imported in the Casava 1.8 paired-end demultiplexed fastq format. Forward reads were truncated at the 280 bp position, and reverse reads at 220 bp, and then both reads were merged. Quality control and feature table construction were achieved using the DADA2 software package and following standard pipelines. Taxonomy was assigned using the SILVA v138.1 database trained on the V3–V4 16S rRNA region with a Naive Bayes classifier. Downstream microbiota data analysis was performed with R software v.4.4.0 using a combination of the ‘phyloseq’ [18], ‘picante’ [19], ‘vegan’ [20], ‘microbiomeR’ [21] and ‘tidyverse’ [22] packages. A thorough explanation of the bioinformatic analysis can be found in the code repository on https://github.com/Gandasegui/hookworm_microbiome.

**Fig. 1:**
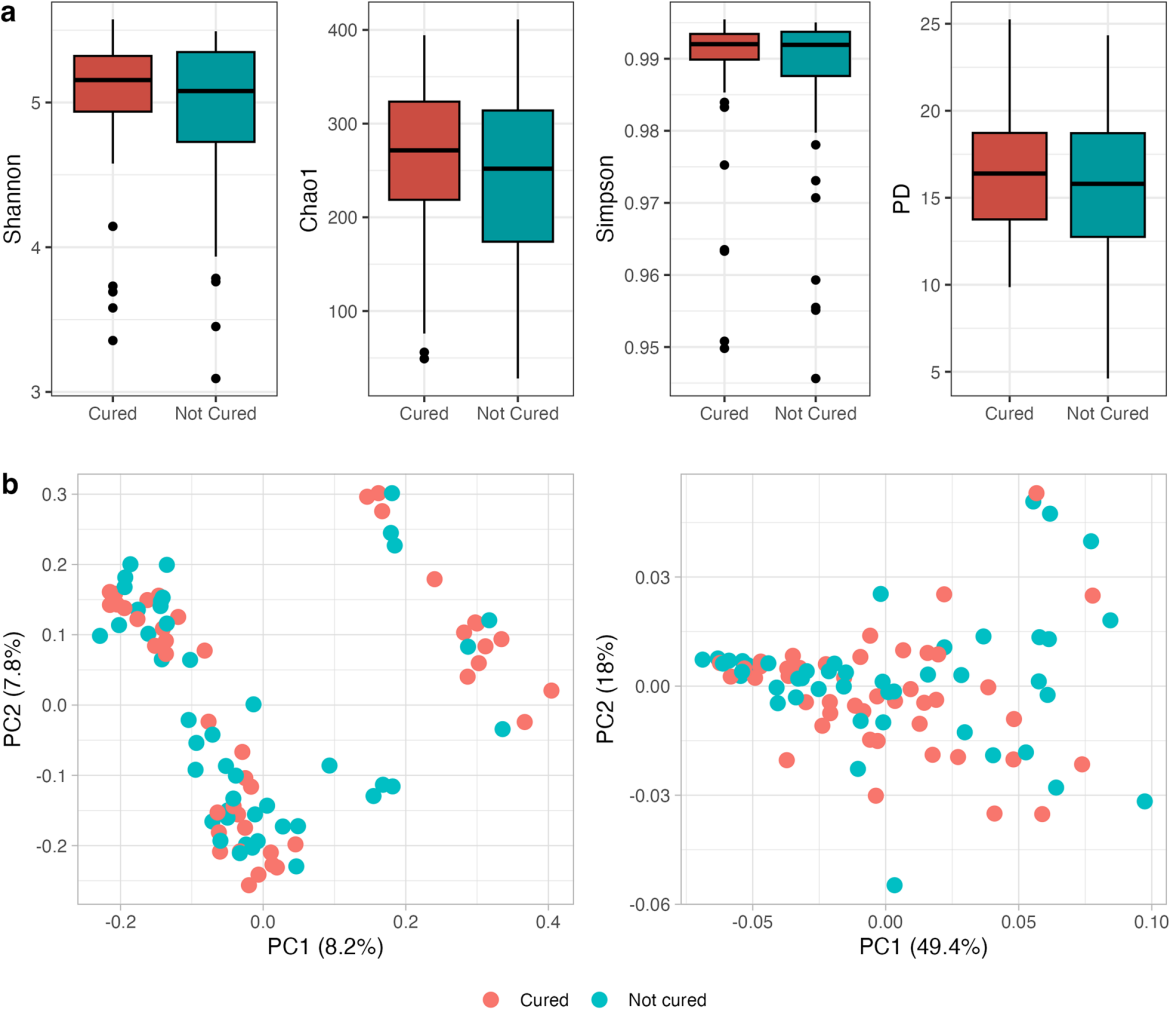
Relationship between alpha diversity and beta diversity of baseline gut microbiota to albendazole efficacy defined as participants who were cured and those who were not cured. **a** Shannon, Chao1, Simpson and PD indices of cured patients and those who were not cured. **b** Relationship between beta diversity and the treatment outcome with albendazole, including unweighted (left) and weighted (right) unifrac principal coordinates analysis. Along with the *y*- and *x*-axis, the percentage of variance explained by PC1 and PC2 is indicated. PC, Principal coordinate; PD, phylogenetic diversity

Alpha and beta diversity were estimated after rarifying at the 1100 sequencing depth, which enabled the inclusion of the 89 samples into downstream analysis. For alpha diversity, we calculated the Shannon index, Chao1, Gini-Simpson and phylogenetic diversity (PD) parameters and we assessed their relationship with ALB efficacy by the Kruskal–Wallis test corrected for multiple testing by the Bonferroni method. Regardless of estimator, no difference in alpha diversity was observed between participants who were cured and those who were not cured (*p*-values for Shannon index, Chao1, Gini-Simpson and PD parameters were 0.48, 0.48, 0.44 and 0.46, respectively) (Fig. 1a). These results suggest that gut microbiota diversity is similar at baseline between individuals that show different responses to the treatment with ALB. We also carried out both unweighted and weighted UniFrac principal coordinates analysis (PCoA) for beta diversity estimation, taking into consideration both cured participants and those who were not cured (Fig. 1b). In the case of unweighted UniFrac PCoA, two distinct clusters of samples are observed. However, this clustering is not associated with the treatment response, as both groups of participants (cured and not cured) are similarly distributed across the clusters. For the weighted analysis, which includes phylogenetic information, no clear structure is observed, with particular attention to principal coordinate 1 (PC1), which explains approximately 50% of the variability among the samples. Thus, no patterns of clustering related to the outcome by PCoA are observed. In summary, these results suggest that, in our samples, either alpha or beta diversity are not associated with ALB efficacy.

We the performed differential abundance (DA) analysis testing at the phylum, family and genus level to explore the possibility of whether changes in the relative abundance of bacteria communities are related to the cure with ALB. For this analysis, we used detection rate and prevalence threshold values of 1% and 10%, respectively. To explore the association of different bacterial communities with the cure with ALB treatment, we used a set of DA testing methods as previously recommended [23]. Differences in the abundance of bacterial communities at the three taxonomic levels were compared between cured participants and those who were not cured using three methods: (i) the conventional Kruskal–Wallis test corrected for multiple testing by Bonferroni; (ii) ALDEx, which is a DA testing tool widely used in microbiome analysis [24–26]; and (iii) Zicoseq, a versatile tool for DA testing that allows confounding factors in the DA analysis to be addressed [27] which, in our study, we included participant sex and age as covariates. No statistically significant differences were observed (*p*-value > 0.05 in all cases; see Additional file 1: Table S1) between the relative abundance of bacteria in cured participants and those who were not cured, among those infected with hookworms. These findings indicate that, in our samples, no specific bacterial communities are associated with ALB efficacy and, consequently, these communities are not modulating drug pharmacokinetics or the host immune system in a way that affects parasite clearance after treatment. Although a recent report suggested that bacteria belonging to the *Clostridia* class could potentially indicate ALB treatment failure in hookworm-infected individuals [13], our analyses, which expand sample size and the robustness of the diagnostic framework, are consistent with those in our previous study that found no association between ALB efficacy and the gut microbiome in participants infected either with *T. trichiura* and hookworms [12].

Beyond conventional statistical tests, we also used supervised and unsupervised ML techniques in this work, as they offer innovative approaches to the analysis of complex microbiome data. Unsupervised ML methods can reveal hidden patterns in the dataset that might not be immediately apparent through traditional analyses [28].

**Fig. 2:**
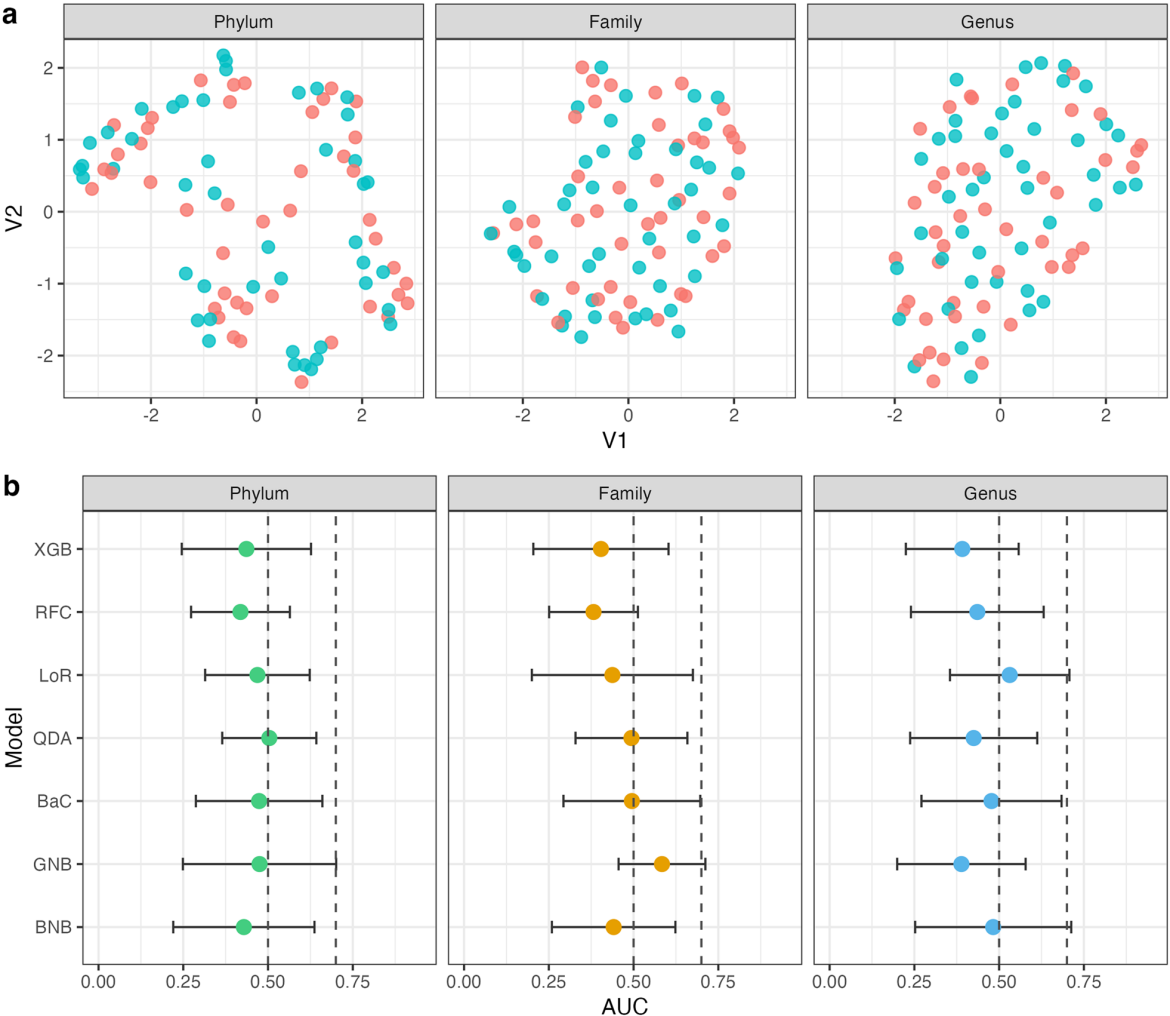
Use of unsupervised and supervised machine learning analysis on relative abundances of bacteria communities at the phylum, family and genus taxonomic levels. **a** Results obtained using the he unsupervised machine learning approach of UMAP on the abundances of bacteria. **b** Predictive capacity of the abundances of bacteria expressed by the AUC on the *x*-axis, and the different models on the *y*-axis (XBG, XGBoost; RFC, Random Forest Classifier; LoR, Logistic Regression; QDA, Quadratic Discriminatory Analysis; BaC, Bagging Classifier; GNB, Gaussian Naive Bayes Classifier; BNB, Bernoulli Naive Bayes Classifier). The dashed lines indicate an AUC of 0.5 and 0.7. AUC, Area under the curve; UMAP, uniform manifold approximation and projection

 The unsupervised ML approach of uniform manifold approximation and projection (UMAP) was used to analyze the abundances of bacteria. These analyses were performed in R using the ‘umap’ package [29]. UMAP is particularly effective in revealing complex patterns and relationships in data. UMAP visualizations were carried out for the relative abundance of bacteria at the phylum, family and genus level to explore the capacity of the bacterial communities to discriminate between cured participants and those who were not cured. As a first step, the relative abundance of bacteria was normalized, then, we assessed the capacity of different groups of bacteria according to the taxonomic level: phylum, family and genus level. Similar to the beta diversity analysis, we did not observe any evidence of clustering of the data based on ALB efficacy regardless of the taxonomic level (Fig. 2a). These UMAP results suggest that the data obtained from this sample set do not exhibit an internal structure based on cured participants and those who were not cured, reinforming the lack of evidence of association between the composition of the gut microbiota and treatment failure.

Relative abundances of bacterial communities at the phylum, family and genus level were also analyzed by supervised ML analysis to assess their predictive value on the treatment response with ALB. In supervised ML, algorithms are trained with features to predict outcomes, enabling them to build models that can make accurate predictions about clinical variables, such as treatment response, by learning from existing data [28]. We evaluated seven models (XGBoost, Random Forest Classifier, Logistic Regression, Quadratic Discriminatory Analysis, Bagging Classifier, Gaussian Naive Bayes Classifier and Bernoulli Naive Bayes Classifier) by tenfold cross-validation with the entire data set using relative abundance as features, at the three taxonomic levels, and “cured” status as the outcome. For each model we calculated the mean area under the ROC curve (AUC) and the standard deviation in the cross-validation. We established a minimum AUC of 0.7 to consider that a model has an acceptable predictive performance. As can be observed in Fig. 2b, the AUC did not exceed 0.7 in any of the models, with the vast majority of models having an AUC < 0.5, indicating a very poor predictive value of the gut microbiota composition for ALB efficacy regardless of the model. Our aim was to evaluate the predictive capacity of relative abundances of bacteria as a marker of potential treatment failure or, more extensively, as a marker of low MDA effectiveness at the population level. However, our data do not exhibit this predictive capability.

Interest in utilizing ML techniques for applied analysis in microbiome research is rapidly increasing. These methods provide powerful tools to identify crucial molecular signatures, uncovering possible patient subgroups and, notably, developing models capable of accurately predicting various phenotypes [30, 31]. The present study represents a comprehensive analysis that integrates ML methods alongside conventional statistical analyses to evaluate not only the correlation of the gut microbiota composition with anthelmintic efficacy, but also its predictive potential. This dual approach is crucial for defining markers of potential treatment failures. Nevertheless, our microbiota data do not exhibit either an association with or a predictive capability for ALB efficacy using the relative abundances at the specified taxonomic levels of bacterial communities as predictive features.

Despite its relatively large sample size and precise diagnostic framework to establish treatment success, our study has a number of limitations. A limited sample size could have also resulted in the lack of association or, particularly, limited the identification of bacterial communities predictive of treatment efficacy. However, similar findings were observed in previous studies, thus supporting the lack of an association between the gut microbiome composition and ALB efficacy. Also, the microbiome profiling conducted through 16S rRNA sequencing captures only a fraction of the microbial community and may occasionally overlook less abundant bacterial taxa that may have biological significance [32]. As noted earlier in this article, our previous study also did not find a clear association between the presence and frequency of three putative benzimidazole resistance SNPs in the beta tubulin gene of *N. americanus* and ALB efficacy [15]. These results, taken together, suggest that other factors, whether originating from the parasite or the host, may be driving the variability in drug response against ALB. We believe that implementing more state-of-the-art, robust and unbiased approaches could help elucidate the interplay between these factors affecting drug efficacy in anthelmintic therapy. Also, further investigations focused on the pharmacomicrobiomics of anthelmintics using different methodologies that allow genomic characterization beyond the ribosomal 16S region, such as shotgun sequencing, might help clarify the role of the gut microbiota in anthelmintic effectiveness. These analyses, coupled with pharmacokinetics data, will contribute to identifying factors that can alter ALB efficacy, ultimately improving therapy and control interventions for STH infections.

## Conclusions

In conclusion, our study cannot establish an association between the composition of the baseline gut microbiota at the time of drug intake and the efficacy of ALB treatment for hookworm infections. Despite employing both conventional statistical analyses and ML techniques, we did not find evidence of a significant correlation or predictive capability between microbiota composition and ALB efficacy. These findings underscore the need for a more comprehensive analytical approach to clarify the role, if indeed present, of the microbiome in anthelmintic efficacy. Future research should include longitudinal studies to track microbiome changes related to the infection and anthelmintics in cured patients, functional metagenomics to understand microbial gene contributions and investigations into host-microbiome interactions to better understand the interaction of the microbiome with the drug. By integrating these advanced methodologies, we can better elucidate the potential influence of gut microbiome on treatment outcomes and develop more effective strategies for STH infection treatment and control. Moving forward, interdisciplinary approaches integrating microbiology, pharmacology, genomics and data science will be essential for advancing our understanding of soil-transmitted helminth infections and optimizing treatment strategies on a global scale. While our study did not yield conclusive insights, it provides a foundation for future investigations aimed at unraveling the intricate interplay between the gut microbiome, the parasite and the host and anthelmintic efficacy.

## Supplementary Information

**Additional file 1. Table S1: P-values for differential abundance testing using Kruskal-Wallis test, ALDEx2, and ZicoSeq at the phylum, family, and genus levels.**

## Data Availability

Participant data cannot be shared publicly because of the need for participants’ consent, but data are available upon reasonable request. The microbiome data used in this study can be found at Sequence Read Archive under the BioProject ID: PRJNA1097510. The code used to analysis the samples can be found on the GitHub public repository. (https://github.com/Gandasegui/hookworm_microbiome).

## Abbreviations

ALB: Albendazole
WHO: World Health Organization
AUC: Area under the curve
DA: Differential abundance
MDA: Mass drug administration
ML: Machine learning
SNP: Single nucleotide polymorphism
STH: Soil-transmitted helminths
UMAP: Uniform manifold approximation and projection
