# Correction: Baseline gut microbiota diversity and composition and albendazole efficacy in hookworm-infected individuals

**DOI:** 10.1186/s13071-024-06619-5

**Published:** 2024-12-20

**Authors:** Javier Gandasegui, Pedro E. Fleitas, Paula Petrone, Berta Grau-Pujol, Valdemiro Novela, Elisa Rubio, Osvaldo Muchisse, Anélsio Cossa, José Carlos Jamine, Charfudin Sacoor, Eric A. T. Brienen, Lisette van Lieshout, José Muñoz, Climent Casals-Pascual

**Affiliations:** 1 Barcelona Institute for Global Health, Hospital Clínic-Universitat de Barcelona, Barcelona, Spain; 2 Manhiça Health Research Centre (CISM), Manhiça City, Mozambique; 3 Mundo Sano Foundation, Buenos Aires, Argentina; 4 Department of Clinical Microbiology (CDB), Hospital Clínic-University of Barcelona, Barcelona, Spain; 5 Leiden University Center for Infectious Diseases, Parasitology Research Group, Leiden University Medical Center (LUMC), Leiden, The Netherlands

**Correction: Parasites & Vectors (2024) 17:387** 10.1186/s13071-024-06469-1

Following publication of the original article [1], it came to the authors' attention that part of the Funding declaration was incorrect. Namely, “ISGlobal acknowledges support from the Spanish Ministry of Science and Innovation through the ‘Centro de Excelencia Severo Ochoa 2019–2023’ Program (CEX2018-000806-S), and support from the ‘Generalitat de Catalunya’ through the CERCA Program.” needed to be corrected to the following: “ISGlobal acknowledges support from the grant CEX2023-001290-S funded by MCIN/AEI/10.13039/501100011033, and support from the Generalitat de Catalunya through the CERCA Program.”

Please be referred to the corrected Funding declaration in the original article. The authors thank you for reading this erratum and apologize for any inconvenience caused.
